# Anaplastic Lymphoma Kinase Is Required for Neurogenesis in the Developing Central Nervous System of Zebrafish

**DOI:** 10.1371/journal.pone.0063757

**Published:** 2013-05-08

**Authors:** Sheng Yao, Mangeng Cheng, Qian Zhang, Mariusz Wasik, Robert Kelsh, Christoph Winkler

**Affiliations:** 1 Department of Biological Sciences and Centre for BioImaging Sciences, National University of Singapore, Singapore; 2 In Vitro Pharmacology, Merck Research Laboratory, Boston, Massachusetts, United States of America; 3 Department of Pathology and Laboratory Medicine, University of Pennsylvania, Philadelphia, Pennsylvania, United States of America; 4 Centre for Regenerative Medicine, Developmental Biology Programme, Department of Biology and Biochemistry, University of Bath, Bath, United Kingdom; Hospital Nacional de Parapléjicos – SESCAM, Spain

## Abstract

*Anaplastic Lymphoma Kinase* (*ALK*) was initially discovered as an oncogene in human lymphoma and other cancers, including neuroblastoma. However, little is known about the physiological function of ALK. We identified the *alk* ortholog in zebrafish (*Danio rerio*) and found that it is highly expressed in the developing central nervous system (CNS). Heat-shock inducible transgenic zebrafish lines were generated to over-express *alk* during early neurogenesis. Its ectopic expression resulted in activation of the MEK/ERK pathway, increased cell proliferation, and aberrant neurogenesis leading to mis-positioning of differentiated neurons. Thus, overexpressed *alk* is capable of promoting cell proliferation in the nervous system, similar to the situation in *ALK*-related cancers. Next, we used Morpholino mediated gene knock-down and a pharmacological inhibitor to interfere with expression and function of endogenous Alk. Alk inhibition did not affect neuron progenitor formation but severely compromised neuronal differentiation and neuron survival in the CNS. These data indicate that tightly controlled *alk* expression is critical for the balance between neural progenitor proliferation, differentiation and survival during embryonic neurogenesis.

## Introduction

*Anaplastic Lymphoma Kinase* (*ALK*) was initially discovered as an oncogene in human anaplastic large cell lymphomas (ALCL), a subset of T-cell lymphomas [1], [2], [3]. Genetic dissection revealed an hybrid gene (NPM-ALK, also known as p80) at the t(2;5)(p23;q35) chromosomal translocation breakpoint, comprising a fusion of a nucleolar protein gene *nucleophosmin* (*NPM*) and a part of a gene coding for ALK, a novel tyrosine kinase. Moreover, another fusion of *alk* with the gene encoding *echinoderm microtubule-associated protein-like4*, encoding EML4-ALK hybrid protein, was identified in a subset of non-small-cell lung cancers (NSCLCs) [4]. To date, several more ALK hybrid proteins have been identified in various cancer types, such as TRK-fused gene (TFG)-ALK, tropomyosin 3 (TPM3)-ALK, tropomyosin 4 (TPM4)-ALK, clathrin heavy chain-like 1 (CLTCL1)-ALK, and moesin (MSN)-ALK [5]. In most, if not all, of these hybrid proteins the distal portion of ALK containing the tyrosine kinase domain is fused with proximal parts of the diverse proteins having oligomerization domains. Consequently, the hybrid proteins undergo spontaneous dimerization, leading to autophosphorylation and ultimately constitutive enzymatic activation of the ALK tyrosine kinase domain.

Overexpression of *ALK* is often observed in human neural tumors, primarily neuroblastoma [6]. Neuroblastomas are embryonic tumors of the peripheral sympathetic nervous system derived from neural crest tissues. It is one of the most frequent pediatric solid tumors, accounting for about 15% of childhood cancer mortality [7]. A clear correlation of *ALK* deregulation with neuroblastoma development has been established [8], [9], [10], [11], and at least ten *ALK* mutations, all found in the tyrosine kinase domain, were identified in the neuroblastoma samples. Among these, R1275Q, F1174L and F1245L/V/C occur most frequently [8], [11]. Increased copy numbers and gene amplifications of the *ALK* locus is also often detected in neuroblastoma patients [8], [10]. These germ line mutations in *ALK* explain most of the inheritable neuroblastomas, but activating mutations can also be somatically acquired. These mutations lead to constitutive autophosphorylation of mutated ALK, which displays increased kinase activity and excess phosphorylation on downstream targets such as Akt, STAT3 and ERK1/2. Mutated *ALK* from neuroblastoma samples is capable of transforming interleukin-3-dependent haematopoietic Ba/F3 cells into cytokine-independent growth [9], as well as NIH3T3 fibroblasts into colony and tumors in nude mice [8]. Knock-down of *ALK* by siRNA [8], [11], or ALK inhibition by small molecules PF-2341066/Crizotinib [10] and TAE684 [9] leads to suppressed cell growth, with decreased proliferation and increased apoptosis in neuroblastoma cells harboring mutated or amplified *ALK*. Together, these studies in neuroblastomas suggest that in a normal cellular context, appropriate *ALK* expression and activity levels need to be maintained for cell proliferation and survival.

While a pathogenic role for *ALK* has been demonstrated for many human cancers, little is known about the physiological function of *Alk* during development [12], [13], [14], [15]. Expression of *ALK* has been reported in murine neural tissues [13], [14], [16] but its function, especially in the CNS, remains elusive. A recent report in chicken showed that Alk controls proliferation of sympathetic neurons in the peripheral nervous system (PNS) (Reiff et al., 2011). In zebrafish (*Danio rerio*), a closely related co-ortholog of Alk, *leukocyte tyrosine kinase* (*ltk*) encodes a 1530 amino acids (aa) long protein [17]. *ltk* is expressed in neural crest cells (NCCs) and iridophores, a pigment cell type widespread in anamniote vertebrates. *ltk* is mutated in zebrafish *shady* mutants, which are characterized by the complete lack of iridophores and the loss of a subset of NCCs by apoptosis. Further analysis revealed that zebrafish *ltk* controls fate specification in a distinct subset of NCCs derived from the multipotent premigratory NCCs [17].

In the present study, we identified the full length zebrafish *alk* sequence and show that *alk* is expressed in the developing zebrafish CNS, that its over-expression results in increased proliferation of neural progenitors and that it is required for neuronal differentiation and survival. This is the first study that investigates Alk function is zebrafish and shows a critical role for Alk in the embryonic CNS.

## Materials and Methods

### Cloning of full-length zebrafish *alk*

The 5′UTR and N-terminal zebrafish *alk* sequences were obtained by 5′RACE (Rapid Amplification of cDNA Ends) using a kit from Invitrogen. The full length *alk* coding sequence was then PCR amplified with Phusion polymerase (Finnzymes), primers ALKatgXhoI (5′- CCGCTCGAGCCACCATGTGTGATAACGCAGCAGAG -3′) and ALKdownXbaI (5′- TGCTCTAGATTACAGCACAGTGGCGTTGT -3′), and cloned into pCS2+ (for capped mRNA preparation) or *alk*:HSE:*cfp* (for generation of transgnic fish, see below) using XhoI and XbaI sites present in these vectors.

### Generation and maintenance of transgenic fish

All animal experiments were performed in accordance with approved IACUC protocols of the National University of Singapore (protocol numbers 075/07; 082/10; BR19/10). Zebrafish inbred AB wild-type strains were crossed to obtain embryos that were raised in 30% Danieau's solution at 28°C and staged as described previously [18]. For generation of transgenic fish, a *alk*:HSE:*cfp* construct was generated that contains the full-length zebrafish *alk* and *cfp* under control of a bidirectional heat-shock promoter in addition to flanking I-*SceI* sites [19], [20]. HSE:*cfp* was used as control. Plasmid DNA and I-*SceI* meganuclease (New England Biolabs) were co-injected into AB wild-type embryos at the one-cell stage as described [21]. On the next day, injected embryos were heat shocked for 1 hr at 39C. Embryos with strong CFP expression were raised to adulthood and crossed to WT AB fish. For screening and maintaining these lines, embryos were heat-shocked after 24 hpf to prevent early embryonic defects caused by *alk* overexpression. F2 or F3 embryos of two independent *alk* overexpressing lines (*alk*:HSE:*cfp* ^1^ and *alk*:HSE:*cfp* ^2^) and the HSE:*cfp* control line were used in the experiments described below.

### Morpholino and RNA microinjection

The following Morpholino oligonucleotides (MO; from GeneTools, Philomath USA) were used: *alk*-ATG MO, 5′-TCCTCTGCTGCGTTATCACACATTC-3′; *alk*-ATG-mismatch control MO, 5′-TCgTCTcCTGCcTTATgACAgATTC-3′ (base substitutions indicated by small letters); splice-site MOs: *alk*-E3I3 MO, 5′-CATTTATGCAGAGCACCTGGTGATG-3′, and *alk*-I3E4 MO, 5′-CAAGGCCCCTGCCAGACAGAATGAT-3′; standard control MO, 5′-CCTCTTACCTCAGTTACAATTTATA-3′; *p53* MO, 5′-GCGCCATTGCTTTGCAAGAATTG-3′. All MOs were injected at 2 mg/ml, except for MO efficiency tests (5 mg/ml) and for the p53 MO (8 mg/ml).

Capped mRNAs were synthesized *in vitro* using the mMESSAGE mMACHINE SP6 Kit (Ambion). *NPM-ALK* mRNA was injected at 50 ng/µl. *alk*ATG-*egfp* mRNA was injected at 50 ng/µl. To test the translation blocking efficiency of the *alk* ATG-MO, a target construct *alk*ATG-*egfp* was generated that contains the *alk* 5′UTR and the ATG-MO target site, in addition to a 6 bp XhoI linker, in-frame with the *egfp* coding sequence.

### RNA *in situ* hybridization, immunostaining and Western blotting

Whole mount RNA *in situ* hybridization and immunostaining were performed as described earlier [22]. An antisense riboprobe comprising the *alk* sequence from nt 6–885 was used to detect *alk* expression, and a sense probe covering the same sequence was used as control. The following primary antibodies were used: anti-phospho-Histone 3 (anti-pH 3; Millipore); anti-HuC/HuD (anti-HuC/D; Molecular Probes); anti-ERK1/2 (Santa Cruz); and anti-phospho-ERK1/2 (anti-pERK; Cell Signaling). For detection, either the VECTASTAIN Elite ABC Kit (Vector Labs) for diamino benzidine (DAB; Sigma) staining was used, or Alexa Fluor-488/568 coupled secondary antibodies (Invitrogen) for fluorescence detection. TUNEL assays were performed using the ApopTag Peroxidase In Situ Apoptosis Detection Kit (Millipore) or with an anti-DIG primary antibody (Roche) for fluorescence detection. For 5-bromo-2-deoxyuridine (BrdU) labelling of cells in S-phase, embryos were incubated in 10 mM BrdU (Sigma) for 15 min on ice before fixation, followed by detection with an anti-BrdU primary antibody (DSHB, University of Iowa) as described previously [23]. For Western analysis, zebrafish embryos were deyolked and proteins extracted as previously described [24]. Western blotting was performed with anti-ERK and anti-pERK antibodies using standard protocols.

### Alk inhibitor treatment

Dechorionated embryos were incubated with the pharmacological Alk inhibitor CEP-26939 [Teva Pharmaceuticals, also known as cmpd 13; 25] at 40 µM with 1% DMSO in Danieau's solution. 1% DMSO in fish medium was used as control. Embryos were treated from 24 hpf to 60 hpf, with changes of medium every 12 hours, and fixed at 60 hpf for further processing.

### Microscopy, cell counting and statistical analysis

Confocal images were taken with a Zeiss LSM 510 Meta laser scanning microscope. All other images were taken with a Nikon SMZ1000 or a Nikon ECLIPSE 90i microscope. pH 3 positive cells were counted in confocal projections of 20 continuous optical sections representing a 50 µm thick area within rhombomeres r4 to r6. For cell counting, BODIPY-Texas-Red (Molecular Probes, Invitrogen) was used to counterstain cell membranes of HuC/D positive neurons. Embryos were stained in 20 µM BODIPY-TR for 1 h prior to imaging [26], [27]. Statistical analysis was done with GraphPad Prism 5. Unpaired two-tailed Student's t-test was used in comparisons between treated and control groups, with p<0.05 indicating statistical significance. Bar graphs were plotted with GraphPad Prism 5, showing means with standard error means (SEM) as error bars.

## Results

### Identification of full-length zebrafish *alk*

A partial zebrafish anaplastic lymphoma kinase (*alk*) sequence was available in the NCBI database (GenBank: XM_686872.2). It is located on chromosome 17 (Ensembl: ENSDARG00000095833) and comprises a putative 4164 base pairs (bp) transcript encoding 1387 amino acids (aa). We used 5′RACE to identify a 216 bp 5′ UTR that contains several STOP codons in all three reading frames, and the predicted translation start site of zebrafish *alk*. The full-length zebrafish *alk* coding region contains 4167 bp encoding a 1388 aa protein. The zebrafish Alk tyrosine kinase (TK) domain (aa 795–1062) has 89.6% aa identity to that of human ALK (aa 1116–1383; GenBank: NM_004304.4). Key tyrosine residues are present in zebrafish Alk (Y957, Y961 and Y962) that correspond to the human ALK kinase domain activation loop, a YXXXYY motif. Notably, this motif presents as YRASYY in all vertebrate Alk proteins, and slightly modified as YRSDYY in the fruit fly [28]. Moreover, three other residues in the TK domain: F1174, F1245 and R1275 that are most frequently affected by gain-of-function mutations in human neuroblastoma [8], [11] are also conserved in zebrafish Alk (F853, F924 and R954, respectively). Together, these data suggest a critical role for the highly conserved Alk residues in zebrafish.

### Zebrafish *alk* expression during embryonic neurogenesis

**Figure 1:**
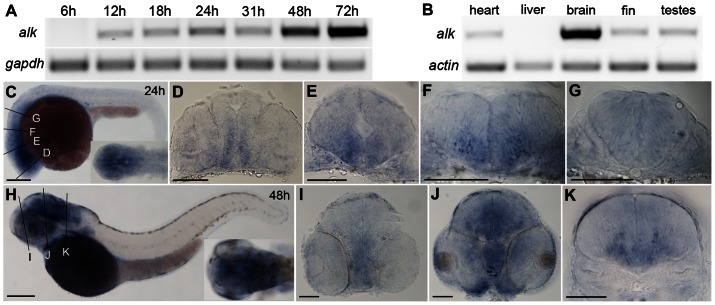
Expression of zebrafish *alk*. **(A)** RT-PCR of cDNA at different embryonic stages. *gapdh* is used as loading control. **(B)** RT-PCR of cDNA from different adult tissues. *beta-actin* is used as loading control. **(C)** Lateral view of whole embryos at 24 hpf with dorsal up and anterior to the left. Insert shows dorsal view of head region. **(D–G)** Transverse sections of embryos at 24 hpf, at forebrain (D), midbrain (E), rhombomere 2 (r2; F) and r5 (G) levels, corresponding to positions indicated by lines in (C). **(H)** Lateral view of whole embryo at 48 hpf with dorsal up and anterior to the left. Insert shows dorsal view of head region. **(I–K)** Transverse sections of embryos at 48 hpf at forebrain (I), midbrain (J), and r5 (K), corresponding to positions indicated by lines in (D). Scale bars: C,H, 200 µm; D–E,I–K, 100 µm.

We examined Alk mRNA transcript expression by RT-PCR at developmental stages from 6 hours post fertilization (hpf) to 72 hpf. *Alk* transcripts are first detected at 12 hpf, and expression levels continuously increased with the most profound increase at 48 hpf (**Fig. 1A**). The appearance of *alk* expression shortly after gastrulation corresponds to the initiation of segmentation and neurulation (10 hpf; [18]. Neurogenesis in the zebrafish hindbrain peaks between 24 hpf and 48 hpf, when the neuron to progenitor ratio changes most significantly [29]. The increase of *alk* mRNA levels from 12 hpf to 48 hpf therefore coincides with neurogenesis in zebrafish. In adult tissues, *alk* is highly expressed in brain, with much lower expression in heart, caudal fin, as well as testis, and none detectable in liver (**Fig. 1B**). Abundant expression of *alk* in adult brain suggests that it may function in adult neurogenesis and neural regeneration, which is prominent in zebrafish [30].

To analyze the spatial control of *alk* expression, whole mount *in situ* hybridization was performed with two antisense *alk* riboprobes representing either the *alk* N- (nt 6–885) or C-terminus (nt 3393–4071) in embryos at 24, 48 and 72 hpf. Both probes showed identical patterns (**Fig. 1C–K** **; Fig. S1; and data not shown**). Sense probes were used as negative control and showed no staining (**Fig. S1**). As early as 24 hpf, *alk* transcripts were broadly expressed in the developing central nervous system (CNS) including the spinal cord, with elevated levels in the brain (**Fig. 1C**). At 48 hpf, strong expression was found in the mid- and hindbrain (**Fig. 1H**), which remained high at 72 hpf (**Fig. S1H,I**). Transverse sections of embryos at 24 (**Fig. 1D–G**) and 48 hpf (**Fig. 1I–K**) at different brain levels showed that *alk* is expressed in the entire brain, with higher expression in ventral than dorsal regions of the early forebrain (**Fig. 1D**) and midbrain (**Fig. 1E**). In the hindbrain, expression levels were higher in regions where differentiated neurons locate rather than in regions with newborn progenitors (**Fig. 1F,K**), especially at 48 hpf [**29**].

### Increased neural proliferation and aberrant neurogenesis in embryos ectopically expressing *alk*

We generated transgenic *alk*:HSE:*cfp* zebrafish lines that simultaneously co-express CFP and wild-type Alk under control of a bi-directional heat-shock promoter (**Fig. S2**)[19]. A control line with only the *cfp* sequence was also established (**Fig. S2A**). Embryos showing no CFP signal after heat shock (transgenic negative siblings (Sib); arrows in **Fig. S2**) were used as negative controls for comparison. *In situ* hybridization at different time points after heat shock at 24 hpf showed elevated levels of *alk* mRNA, detectable as soon as 1 hour post heat shock (hph; **Fig. S3A**), degraded over time (**Fig. S3B**), and eventually returned to the baseline *alk* concentration at 22 hph (**Fig. S3C**). A second heat shock performed at 48 hpf reactivated the transgene (**Fig. S3D**). CFP negative embryos showed no additional *alk* RNA (arrows in **Fig. S3**), confirming that they were transgenic negative siblings. Notably, after a 1.5 hour heat shock at 39.5°C at 10.5 hpf, all transgenic *alk* expressing embryos in both lines showed marked morphological malformations of the brain (**Fig. S2D,E**), similar to embryos injected with human *NPM-ALK* mRNA (data not shown). In contrast, HSE:*cfp* control embryos showed no defects with the same treatment (**Fig. S2C**), suggesting that the observed defects are caused by overexpressed Alk.

**Figure 2:**
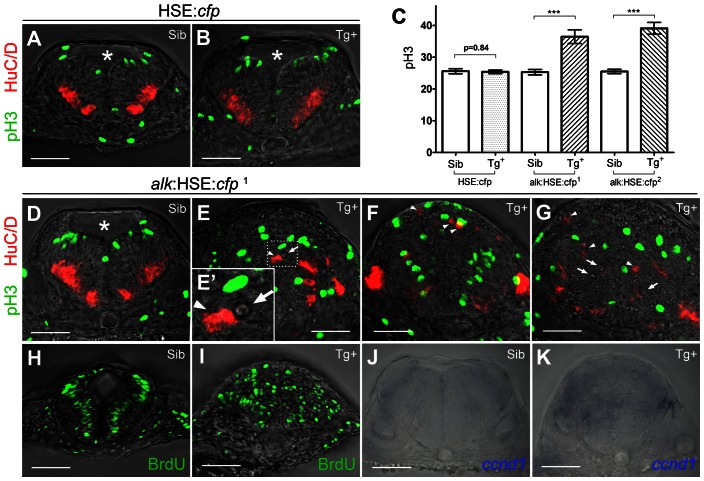
Overexpression of *alk* promotes cell proliferation and affects neurogenesis. **(A,B)** Confocal sections of HSE:*cfp* embryos. Sib (A) and Tg+ (B) embryos show no difference in number and distribution of pH 3 and HuC/D positive cells. **(C)** Y-axis indicates numbers of pH 3 positive cells counted in a 50 µm confocal stack of the hindbrain. Mean ± SEM, n = 10 embryos in each group. Sib and Tg+ HSE:*cfp* embryos were not significantly different (p = 0.84). Sib and Tg+ embryos of both *alk*:HSE:*cfp* lines were significantly different (***p<0.001). Unpaired two tailed t-test. **(D–G)** Confocal sections of *alk*:HSE:*cfp* ^1^ embryos. Sib (D) and Tg+ (E–G, from three different embryos) had different neural tube shapes. Dividing cells (pH 3, green) and neurons (HuC/D, red) in Tg+ embryos (E–G) were mispositioned (arrowheads), with aberrant patterns. **(E′)** High magnification of the boxed area in (E). Asterisk labels 4^th^ ventricle. Arrows label small cavities found in the neural tube. **(H,I)** Confocal sections of *alk*:HSE:*cfp* ^1^ embryos, with BrdU labelled cells in S-phase. In Sib (H), BrdU positive cells occupy a region between dividing cells and neurons that exited the cell cycle, in a pattern complementary to pH 3 and HuC/D in (A,B,D). In Tg+ (I), BrdU positive cells were randomly positioned. Smaller dimension of samples in (H,I) might be due to HCl treatment in the experiment procedure. **(J,K)** Manual sections of embryos after *in situ* hybridization showed expanded *ccnd1* expressions in Tg+ (K) in comparison to Sib embryos (J). Sib, transgenic negative siblings. Tg+, transgenic positive embryos. All images are sections perpendicular to neural tube. Scale bars: 50 µm.

Defects in embryos heat shocked at 10.5 hpf were investigated at 24 hpf. For quantification of cell numbers in the experiments outlined below, we analyzed the hindbrain between rhombomeres r4 and r6, using the otic vesicles as landmarks in transverse sections (**Fig. 2**). Importantly, similar findings were also observed in other regions of the CNS where endogenous *alk* is expressed at 24 hpf (**Fig. 1C**; data not shown). In *alk*-overexpressing embryos (Tg+), the 4^th^ ventricle, a normally T-shaped opening in the hindbrain (asterisks in **Fig. 2A,B,D**), was completely absent. Instead, several small cavities were observed (arrows in **Fig. 2E,G**, with high magnification in the inset **E′**). This suggested early neural tube defects, since the 4^th^ ventricle, like other brain ventricles, is formed by proliferation and differentiation in the early neuroepithelium [18]. Accordingly, proliferation is abundant at this stage in the zebrafish hindbrain [29], and cell divisions occur almost exclusively at the most apical ventricular surface [29], [31]. In control siblings, proliferating cells positive for phospho-histone 3 (pH 3) were observed at the apical surface as expected (**Fig. 2A,B,D**). In contrast, in all *alk* Tg+ embryos of the *alk*:HSE:*cfp* ^1^ line, pH 3 positive cells were localized in a completely randomized manner (**Fig. 2E–G**). Similarly, differentiated postmitotic neurons are normally found in two clusters at each side of the basal and lateral regions of the neural tube (arrowheads in **Fig. 2A,D** **;** [29]. In *alk* Tg+ embryos, HuC/D positive cells showed ectopic appearance at places, where they never occur in controls (**Fig. 2E–G**), while the patterns of pH3^+^ and HuC/D^+^ cells was not affected in HSE:*cfp* Tg+ embryos (**Fig. 2B**). To rule out possible positional effects of the inserted transgene, embryos of the second line *alk*:HSE:*cfp* ^2^ were examined and showed the same results (**Fig. S4B**).

Counting pH 3 positive cells revealed that Tg+ embryos of both *alk*:HSE:*cfp* ^1^ and *alk*:HSE:*cfp* ^2^ lines had an average of 44% and 53% more cells in M-phase compared to their Sib control groups, respectively (36.50 ± 2.16 vs. 25.30 ± 0.84; 39.10 ± 1.87 vs. 25.50 ± 0.69) (**Fig. 2C**). Consistently, HSE:*cfp* control embryos showed no significant difference from Sib embryos (25.40 ± 0.54 vs. 25.60 ± 0.81). This indicates that overexpression of *alk* but not CFP significantly elevates cell proliferation in the hindbrain. DNA synthesis in cells at S-phase was analyzed by 5-bromo-2-deoxyuridine (BrdU) labelling. In controls, BrdU cells occupy intermediate domains between the ventricular surface division zones and areas rich in postmitotic neurons, in a pattern complementary to pH 3 and HuC/D positive cells (**Fig. 2H**). In *alk*:HSE:*cfp* ^1^ Tg+ embryos, S-phase cells were not restricted to these positions but scattered throughout the hindbrain (**Fig. 2I**). The total area with cells in S-phase was drastically expanded, which made cell counting impossible. Interestingly, an increase of cells in S-phase has also been reported in primary sympathetic ganglia neuron cultures from embryonic chicken transfected with human *ALK* gene [15].

To test whether the observed increased proliferation is associated with upregulation of the cell cycle machinery, we evaluated the expression of *cyclin D* (*ccnd1*), which is known to promote proliferation by regulating cyclin dependent kinases CDK4 and CDK6 [32]. *cyclin D* is overexpressed in many cancers [32] including *ALK* positive neuroblastomas [5]. In non-transgenic control embryos, *ccnd1* expression was observed in two stripes of the dorsal hindbrain (**Fig. 2J**). In contrast, *alk*:HSE:*cfp* ^1^ Tg+ embryos exhibited an expansion of *ccnd1* expression into other regions of the hindbrain (**Fig. 2K**).

### Overexpression of *alk* activates MAPK signaling *in vivo*

**Figure 3:**
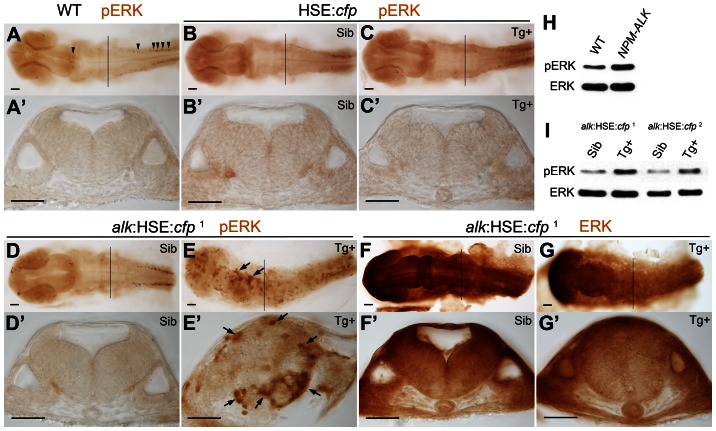
MAPK signaling is activated upon *alk* overexpression. **(A,A′)** phospho-ERK (pERK) immunostaining of a 24 hpf wild-type embryo without heat shock. Endogenous MAPK activation is evident in the caudal most hindbrain and spinal cord, but low in other parts of the CNS. **(B,B′,C,C′)** Both Sib (B,B′) and Tg+ embryos (C,C′) of the HSE:*cfp* control line were normal and no additional pERK was observed. **(D,D′,E,E′)** Ectopic pERK (arrows) was evident in Tg+ (E,E′) of the *alk*:HSE:*cfp* ^1^ line compared to Sib (D,D′). **(F,F′,G,G′)** Total ERK was ubiquitously distributed in both Sib (F,F′) and Tg+ (G,G′) embryos of the *alk*:HSE:*cfp* ^1^ line and showed no difference. **(A′–G′)** Transverse sections from embryos at r5 level as indicated by black lines in (A–G). Sib, transgenic negative siblings. Tg+, transgenic positive embryos. ERK, total ERK1/2. pERK, phosphorylated-ERK1/2. Scale bars: 50 µm. **(H)** Western blot analysis showing more pERK protein in *NPM-ALK* mRNA injected embryos than in WT at 11 hpf. Each lane represents protein content from five embryos of a 20–50 embryo pool. **(I)** Western blots showing increased pERK levels in Tg+ versus Sib embryos at 22 hpf in both *alk*:HSE:*cfp* lines. Each lane contains proteins equivalent to five embryos of a 20–50 embryo pool. Only dissected anterior parts of embryos were used to eliminate endogenous pERK originating from spinal cord and tail bud.

Activation of several signaling pathways has been reported in human *ALK*-related cancers including MEK/ERK pathway involved in cell proliferation [5]. In *Drosophila*, *Alk* functions through ERK activation during visceral mesoderm development [33], [34], [35], [36]. To test whether the MEK/ERK pathway is affected by the *alk* overexpression in zebrafish, we examined phosphorylation of ERK1/2 (pERK) and expression of total ERK levels using anti-pERK and anti-ERK antibodies, respectively (**Fig. 3** **; Fig. S4**).

In 24 hpf WT embryos without heat shock, increased pERK concentration could only be found in forebrain, mid-hindbrain boundary, caudal-most hindbrain, spinal cord (**Fig. 3A**), as well as the tail bud (image not shown). There were only few individual pERK positive cells in the hindbrain (**Fig. 3A**). This distribution was found in both Sib and Tg+ embryos of the HSE:*cfp* line, with no additional pERK signals (**Fig. 3B,C**). In cross sections at the level of r5, usually no pERK positive cells could be seen (**Fig. 3A** **′,B′,C′**). This demonstrated that neither heat shock nor overexpression of CFP activated ERK phosphorylation. In contrast, in Tg+ embryos of *alk*:HSE:*cfp* lines, pERK was strongly expressed across the entire CNS albeit in a mosaic pattern (**Fig. 3E** **,E′; Fig. S4D,D′**), while the total ERK concentration and distribution were similar to the control Sib line (**Fig 3F** **,F′,G,G′**). Consistently, Western blot analysis showed increased pERK levels but comparable ERK expression in Tg+ *alk*:HSE:*cfp* embryos at 22 hpf when compared to Sib controls (**Fig. 3H**). The fusion protein NPM-ALK is a known highly oncogenic form of ALK capable of activating MEK-ERK pathway (Chiarle et al., 2008). Accordingly, embryos at 11 hpf injected with human *NPM-ALK* mRNA showed increased pERK compared to WT controls, with similar levels of total ERK protein (**Fig. 3H**). Taken together, overexpression of *alk* causes up-regulation of pERK while total ERK levels remain unchanged. Ubiquitous MEK/ERK activation in the CNS upon *alk* overexpression is consistent with the observed deregulation of cell proliferation and aberrant neurogenesis.

### Increased apoptosis but normal cell proliferation in the hindbrain after *alk* knock-down

Morpholino oligonucleotides capable of blocking zebrafish *alk* translation (ATG-MO) and a pair of splice blocking MOs (Spl-MO) were designed to knock-down *alk* (**Fig. S5A**). As shown in **Fig. S5B**, the *alk* ATG-MO suppressed GFP expression from an *alk*ATG-*egfp* target construct, while a corresponding *alk* ATGmismatch-MO with 5 nt substitutions failed to block GFP expression (**Fig. S5C–F**). Two splice MOs were designed to prevent splicing of intron 3, which results in a 289 bp insertion leading to frame shift with nine possible premature STOP codons (**Fig. S5G**). RT-PCR with RNA from *alk* Spl-MO injected embryos at 24 hpf showed a size increase as expected (**Fig. S5H**).

**Figure 4:**
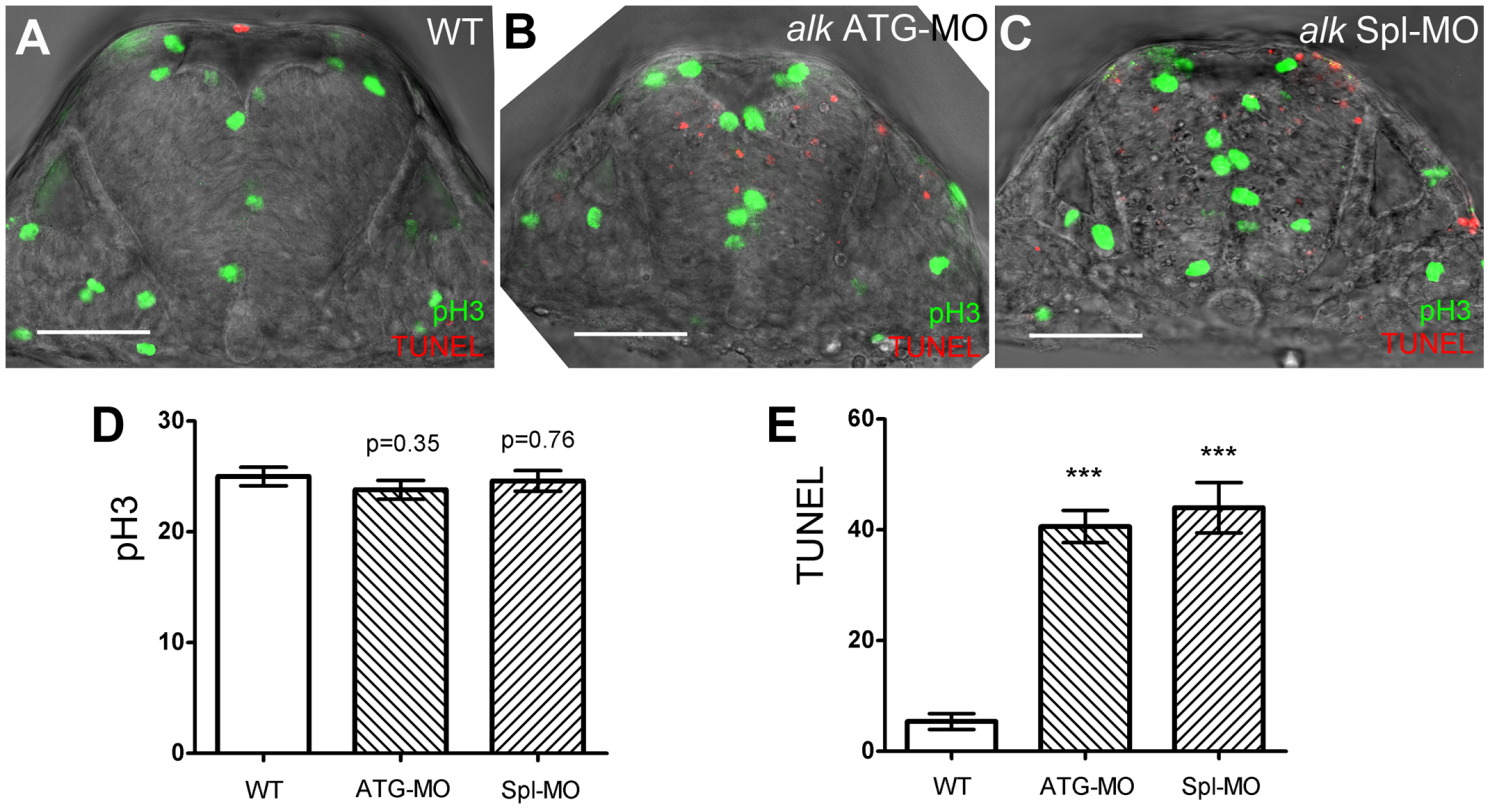
Knock-down of *alk* does not affect proliferation but induces apoptosis in the hindbrain. **(A–C)** Confocal sections of 22 hpf embryos after immunostaining with pH 3 (green) and TUNEL (red). Compared to WT (A), both *alk* ATG-MO injected (B) or *alk* Spl-MO injected (C) embryos show pH 3 positive cells at normal positions, but more TUNEL positive cells are evident. Note that the size of neural tube in (B,C) is smaller than in (A). **(D)** Y-axis indicates numbers of pH 3 positive cells in a 50 µm thick confocal stack of hindbrain. Numbers are not significantly different in *alk* ATG-MO or *alk* Spl-MO from WT. **(E)** Y-axis indicates numbers of TUNEL positive cells in the same samples. Both *alk* ATG-MO and *alk* Spl-MO numbers were different from that in WT with high significance (***p<0.001). Mean ± SEM, n = 5 embryos in each group. Unpaired two tailed t-test. Scale bars: 50 µm.

*alk* ATG-MO or Spl-MO were injected into zebrafish embryos at the one- or two-cell stage, and apoptosis was examined at 22 hpf using a transferase mediated dUTP nick end labeling (TUNEL) assay. This showed increased apoptosis in the CNS (**Fig. 4B,C,E** **;** **Fig. S6C,F**) when compared to uninjected WT (**Fig. 4A**; **Fig. S6A**). Also, the size of the neural tube appeared smaller after *alk* knock-down when compared to WT (**Fig. 4A**). Neither a standard control MO nor the *alk* ATGmismatch-MO resulted in increased apoptosis (**Fig. S6B,E**), suggesting that the increased apoptosis was specific to *alk* deficiency. To further exclude MO off-target effects due to p53 activation [37], a p53 MO [38] was co-injected with *alk* MOs. This did not rescue the apoptosis defects caused by *alk* ATG-MO or *alk* Spl-MO (**Fig. S6D,G**) indicating that the *alk* MO knock-down effects are specific. Thus, *alk* depletion seems to promote apoptosis in the CNS independent of the p53 pathway. Interestingly, increased apoptosis was almost exclusively observed in the CNS, where endogenous *alk* is expressed broadly during early stages (24 hpf; **Fig. 1C**) thus further supporting the specificity of the observed defects.

We also tested whether *alk* inhibition affected cell proliferation. In embryos injected with either *alk* ATG-MO or Spl-MO, pH 3 positive cells were positioned normally (**Fig. 4B,C**). Counting of pH 3 positive cells within the r4 - r6 hindbrain region (**Fig. 4D**) revealed that neither *alk* ATG-MO (23.80 ± 0.86) nor *alk* Spl-MO (24.60 ± 0.93) resulted in a significant difference when compared to WT (25.00 ± 0.84). In contrast, within the same samples, counting of TUNEL positive cells (**Fig. 4E**) showed that both *alk* ATG-MO (40.60 ± 2.93) and *alk* Spl-MO (44.00 ± 4.56) injected embryos had significantly increased number of apoptotic cells as compared to controls (5.40 ± 1.44). These findings suggest that *alk* deficiency may not affect dividing cells in M-phase. Since the majority of cell divisions at this stage represent proliferative neural progenitors, the increased apoptosis observed in *alk* morphants most likely does not affect these progenitor cells, but rather other, possibly more mature neuronal cell types.

### Knock-down of *alk* impairs neuronal differentiation and reduces neuron numbers in the hindbrain

**Figure 5:**
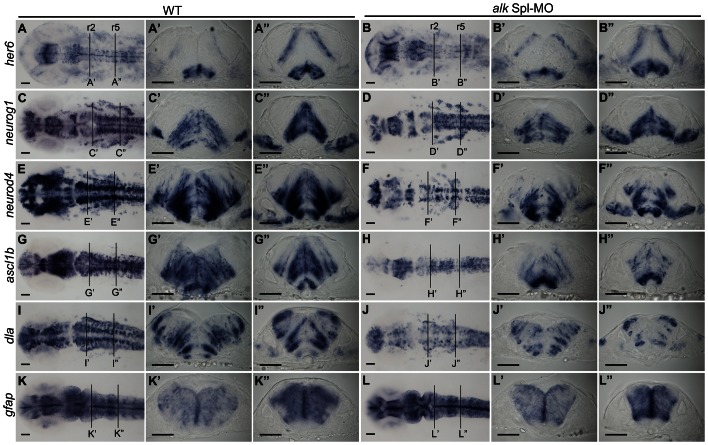
Knock-down of *alk* impairs neuronal differentiation. **(A–L)** *In situ* hybridization of neuronal marker genes with wild-type (A,C,E,G,I,K), and *alk* Spl-MO injected embryos (B,D,F,H,J,L) at 22 hpf. Images in first row show dorsal views of head region with anterior to the left. **(A′–L′,A″ –L″)** Transverse cross sections at the level of r2 (A′–L′) and r5 (A″ –L″) in WT embryos or *alk* Spl-MO injected morphants. **(A,A′A″,B,B′B″)** *her6* expression was unchanged in *alk* morphants compared to WT. **(C,C′,C″,D,D′,D″)** *neurog1* expression was unchanged or only slightly reduced in morphants. **(E,E′,E″,F,F′,F″)** *neurod4* expression was strongly reduced in morphants in several regions including the hindbrain. **(G,G′,G″,H,H′,H″)** Similarly, *ascl1b* expression in *alk* morphants was also significantly reduced. **(I,I′,I″,J,J′,J″)** *dla* expression in *alk* morphants was also reduced. **(K,K′,K″,L,L′,L″)** Glia marker *gfap* expression was unchanged. Scale bars: 50 µm. Lines at r2 and r5 indicate levels of cross sections.

As direct targets of Notch signaling, *Hes* genes (*Hes1* and *Hes5* in mammals) are abundantly expressed in undifferentiated neural progenitors and essential for maintaining stem cell identity [39]. We found no expression change of *hairy-related 6* (*her6*), the zebrafish *Hes1* homolog [40], in *alk* morphants (**Fig. 5B** **,B′,B″; Fig. S7B**) suggesting that proliferation of undifferentiated neural progenitors is not affected by *alk* knock-down. In differentiating neural cells, proneural gene expression is up-regulated as Notch-Hes signaling is switched off. Combined expression of different proneural genes restricts neuronal lineages, forces cell cycle exit, initiates neuronal differentiation and promotes neuron survival [41], [42], [43]. Expression of several zebrafish proneural genes was analyzed to study the effect of *alk* knock-down on neuronal precursor differentiation. *Neurogenin 1* (*neurog1*, *neurod3*) expression was unchanged or only slightly reduced in *alk* morphants (**Fig. 5D** **,D′,D″; Fig. S7D**). On the other hand, expression of the zebrafish *atonal* ortholog, *neurogenic differentiation 4* (*neurod4*, *zath3*) was significantly reduced throughout the CNS, including the hindbrain (**Fig. 5F** **,F′,F″; Fig. S7F**). The *achaete-scute complex like* (*ascl* or *ash*) genes belong to another proneural gene family that can act independently from *atonal*-related genes in vertebrates [44]. Expression of *ascl1b* was also reduced in *alk* morphants (**Fig. 5H** **,H′,H″; Fig. S7H**). Moreover, *delta A* (*dla*), a Notch ligand highly expressed in differentiating neurons, was also reduced (**Fig. 5J** **,J′,J″; Fig. S7J**). Finally, *glial fibrillary acidic protein* (*gfap*) expression was not changed in *alk* morphants (**Fig. 5L** **,L′,L″; Fig. S7L**). Of note, *gfap* is expressed in radial glia cells that overlap with neural progenitors at this embryonic stage. All of the genes mentioned above were expressed normally in *alk* ATGmismatch-MO injected embryos (**Fig. S7A,C,E,G,I,K**). Taken together, we observed a down-regulation of several proneural genes in *alk* morphants. At the same time, progenitor marker expression was unchanged and cell proliferation was not altered. This indicates that progenitor cells do not require *alk* activity. Instead, we propose that *alk* knock-down affects early neuronal precursors that fail to differentiate and instead undergo programmed cell death.

**Figure 6:**
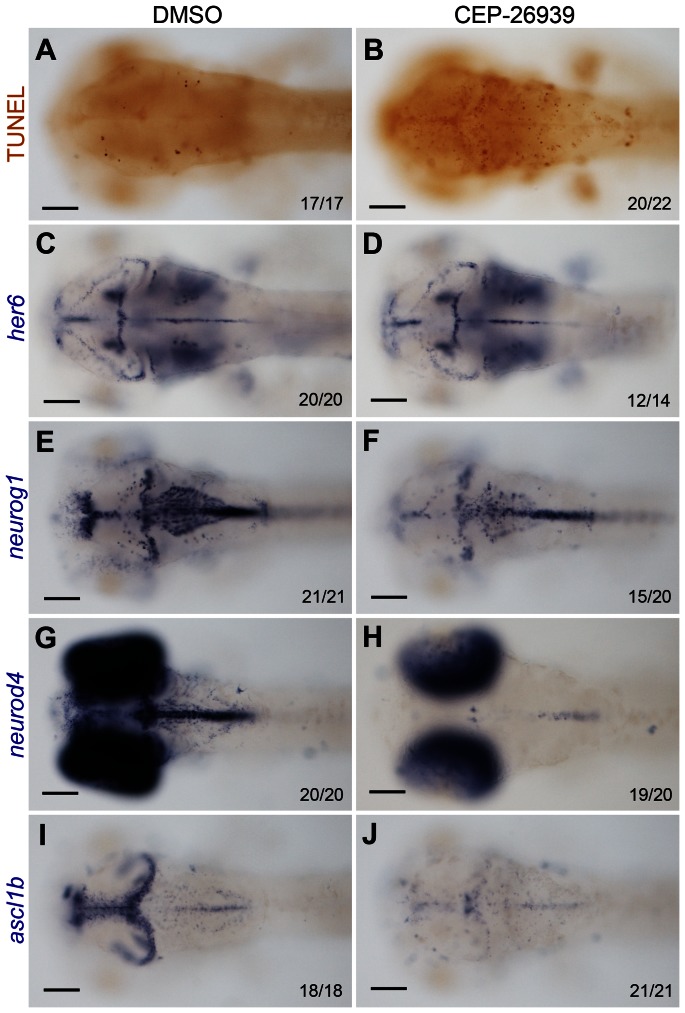
Inhibition of Alk activity by CEP-26939 phenocopies *alk* morphant defects. **(A,C,E,G,I)** Embryos at 60 hpf treated with 1% DMSO from 24 hpf were used as negative controls. **(B,D,F,H,J)** 60 hpf embryos treated with 40 µM CEP-26939 from 24 hpf. **(A,B)** Inhibitor treated embryos showed more TUNEL-positive apoptotic cells in mid- and hindbrain (B) when compared to DMSO treated embryos (A). **(C,D)** No significant change was observed for *her6* expression in inhibitor treated embryos. **(E,F)** *neurog1* was only slightly reduced in inhibitor treated (F) compared to DMSO treated embryos (E). **(G,H,I,J)** Both *neurod4* and *ascl1b* expression domains in mid- and hindbrain were almost completely absent in inhibitor treated embryos (H,J). All images show dorsal views with the focal plane on mid- and hindbrain, with anterior to the left. Numbers in each image indicate individuals similar as that shown as representatives and total numbers of embryos investigated. Scale bars: 100 µm.

MOs elicit their effects from earliest embryonic stages onward. To achieve *alk* deficiency at later embryonic stages, a pharmacological ALK inhibitor, CEP-26939 was used. Both CEP-26939 and its closely related analog ALK inhibitor, CEP-28122, have been reported to lead to growth inhibition in a variety of cultivated human ALK-related cancer cell lines, including neuroblastoma, through caspase 3/7 activation and apoptosis [25], [45]. In our experiment, zebrafish embryos were incubated in fish medium containing CEP-26939, or DMSO alone as a negative control, starting at 24 hpf. Embryos were fixed at 60 hpf and examined for *her6*, *neurog1*, *neurod4* and *ascl1b* expression, as well as apoptosis (**Fig. 6**). Similar to the findings in *Alk* morphants, TUNEL staining revealed increased apoptosis in the CNS of the ALK inhibitor treated embryos (**Fig. 6A,B**), which was also confirmed by Acridine Orange staining in living embryos at 48 hpf (data not shown). Consistent with the MO experiments, no difference in *her6* expression was seen between inhibitor- and DMSO-treated groups (**Fig. 6C,D**). Furthermore, only a weak reduction of *neurog1* expression was observed with no major change in its overall pattern (**Fig. 6E,F**). On the other hand, expression of *neurod4* and *ascl1b* was strongly reduced and almost completely absent in the mid- and hindbrain in CEP-26939 treated embryos (**Fig. 6G,H,I,J**). Significantly, in all regions where *neurog1*, *neurod4* or *ascl1b* expression was reduced, the number of apoptotic cells was strongly increased. Thus, this pharmacological ALK inhibitor fully recapitulated the effects observed after MO-mediated ALK depletion providing further evidence that ALK activity is required for the differentiation and survival of early neural precursors in the developing CNS.

**Figure 7:**
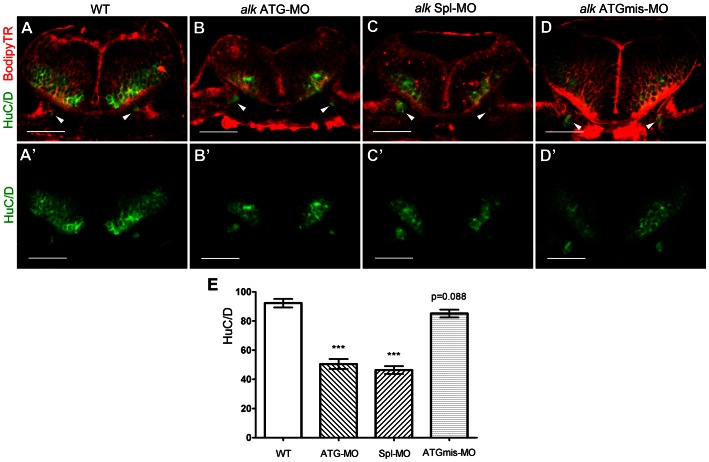
Knock-down of *alk* reduces the number of differentiated hindbrain neurons. **(A–D)** Confocal sections of 31 hpf embryos at r5 level immunostained with the pan-neuronal marker HuC/D (green) and co-stained with bodipy-Texas Red (TR, red). The HuC/D channel (green) is separately shown in (A′–D′). Arrowheads point to emerging neurons in the ear, serving as landmark to indicate that all sections were at the same position. In both *alk* ATG-MO (B,B′) or *alk* Spl-MO (C,C′) injected embryos, the areas of postmitotic neurons were clearly smaller than in WT (A,A′) or *alk* ATGmismatch-MO injected embryos (D,D′). The proliferative ventricular and subventricular zones were normal in size. **(E)** Y-axis indicates numbers of HuC/D expressing cells on sections. Both *alk* ATG-MO and *alk* Spl-MO groups were significantly different from WT (***p<0.001), whereas the change in *alk* ATGmismatch-MO group was not significant (p = 0.088). Mean ± SEM, N = 12 in WT and ATG-MO, N = 14 in Spl-MO and ATGmismatch-MO. Unpaired two tailed t-test. Scale bars: 50 µm.

The observed reduction of proneural gene expression suggested compromised neuronal differentiation and survival. To test to what degree *alk* deficiency affects the final steps of neurogenesis, i.e. the formation of fully differentiated neurons, expression of the pan-neuronal marker HuC/D [46], [47] was analyzed. In the hindbrain at 31 hpf, areas where postmitotic HuC/D+ neurons are located were significantly reduced in size in *alk* morphants (**Fig. 7B** **,B′,C,C′**) when compared to WT (**Fig. 7A** **,A′**) or ATGmismatch-MO injected embryos (**Fig. 7D** **,D′**). In contrast, proliferative ventricular and subventricular zones appeared normal, when BODIPY-Texas Red was used to label cells (**Fig. 7E**), an average of 92.25 ± 2.94 HuC/D positive neurons was counted per confocal single plane in controls (representing a 5 µm optical section). Cell numbers in the *alk* ATGmismatch-MO injected group (85.14 ± 2.71) were not significantly different from WT. In contrast, numbers of HuC/D positive neurons were reduced to 50.50 ± 3.43 in *alk* ATG-MO injected embryos, and 46.36 ± 2.64 in *alk* Spl-MO injected embryos. In conclusion, *alk* ATG-MO or *alk* Spl-MO injected embryos show a significant reduction in the number of differentiated neurons in the hindbrain when compared to WT. Thus, *alk* is required for differentiation and survival or early neural precursors in order to maintain the correct number of fully differentiated hindbrain neurons at later stages.

## Discussion

This is the first study reporting structure and functional characterization of zebrafish *alk*. It is also the first analysis of the physiological role of *Alk* in the vertebrate central nervous system (CNS). We show that zebrafish *alk* is expressed in the developing CNS and is required for differentiation and survival of neural cells. The full length zebrafish *alk* transcript encodes a deduced 1388 amino acids (aa) protein that matches a predicted partial zebrafish *alk* sequence in the NCBI database (GenBank: XM_686872.2), except an additional Glutamine (Q1280) in a Q_(13)_ repeat located at the C-terminal end, and a Serine (S309) to Alanine (A309) replacement in a S_(4)_ repeat located in its N-terminus. These differences most likely reflect polymorphisms between different zebrafish wild-type strains. Interestingly, the N-terminus of zebrafish Alk (aa 1-708) shares only 25.3% aa identity with its human counterpart (aa 1-1029) and seems to be significantly shorter, while the TK domain shows 89.6% identity. Similarly, chicken Alk (GenBank: XM_419364.3) also has a conserved TK domain (aa 970–1237; 95.5% identical to human), but a much shorter N-terminus (aa 1–883) with only 53.3% identity. When Alk sequences from 17 vertebrate species and *Drosophila melanogaster* were compared, the TK domain was the most conserved part among all Alk's. In contrast, the Alk N-terminus showed much more variation, consistent with the idea that the N-terminus of Alk has undergone extensive divergence during vertebrate evolution. In mammals, ALK and LTK can be clearly distinguished by their extracellular domains, with respect to its length (1030 aa vs. 424 aa) and the presence or absence of MAM domains, respectively [48], [49]. Phylogenetic analyses suggested that the MAM domain is a feature of a common Alk/Ltk ancestor, and has been retained in Ltk in zebrafish and chicken but not mammals [17]. It is likely that Ltk and Alk evolved their N-termini differently in mammals and in teleosts. As a consequence, mammalian Ltk lost some of the fish specific Ltk functions, such as for iridophore development [17].

### Expression of *alk* in the central nervous system

In mouse, *Alk* is prominently expressed in the diencephalon, the neuroepithelium surrounding the fourth ventricle, the ventricular and subventricular zone of the cortex, the midbrain, and the medulla oblongata from day 10.5 to day 16.5 [14]. Similar to mouse, *alk* is expressed in most of the central nervous system (CNS) in zebrafish. However, in mouse and chicken, *Alk* expression is also detected in the spinal cord [12], [14]. In contrast, zebrafish *alk* could not be detected in the spinal cord under the experimental conditions used, especially after 24 hpf (**Fig. 1C,H**). This could possibly reflect species-specific differences in *Alk* expression among different vertebrates. In mouse, rat, and chicken, *Alk* is also expressed in the peripheral nervous system (PNS) including the trigeminal ganglia, facial ganglia, vestibulocochlear ganglia, inferior ganglia of the vagus nerve, as well as dorsal root ganglia and sympathetic ganglia [12], [13], [14]. In our study, we could not detect expression of zebrafish *alk* in the PNS. On the other hand, the similar spatiotemporal expression patterns of *Alk* in the early CNS in mouse [**14**], chicken [12], and zebrafish (this study) suggest that the activity and function of the highly related Alk tyrosine kinase domain is conserved during CNS neurogenesis.

### *In vivo* activity of over-expressed, non-mutated Alk

Receptor protein tyrosine kinases (RTKs) can undergo spontaneous dimerization, transphosphorylation of monomers, and activation. This process is promoted and stabilized by the presence of RTK ligands [50], [51]. In our *alk* overexpression experiments, Alk proteins could have spontaneously formed active dimers, resulting in RTK activation. Such autophosphorylation of ALK in absence of any ligand was also reported in cell culture [52]. The observed activation of MEK/ERK pathway confirmed that the transgenic Alk proteins are expressed and enzymatically active. Interestingly, we observed a high degree of mosaicism of MEK/ERK activation although the Alk transgene was ubiquitously expressed. While the exact reason for this remains unclear, it suggests a dynamic regulation of intracellular signaling when Alk is activated in this *in vivo* context. Overexpression of *alk* in these transgenic lines resulted in enhanced cell proliferation with increased numbers of cells in M-phase and up-regulation of *ccnd1* expression. Furthermore, aberrant neurogenesis patterns were observed with ectopic localization of differentiated neurons. We therefore propose that ectopic activation of Alk leads to excess MAPK activity resulting in increased proliferation of neuronal progenitors, randomized cell cycle exit and differentiation, and consequently mis-positioning of differentiating neurons. Triggering *alk* overexpression at a later stage (24 hpf) also enhanced proliferation but strikingly to a much lesser extent and in a spatially more restricted fashion (data not shown) when compared to overexpression at 10.5 hpf (**Fig. 2**). Recent studies suggested that overexpression of mutated *ALK* works cooperatively with *MYCN* overexpression to promote the severity of induced neuroblastoma [53], [54]. Interestingly, zebrafish *nmyc1*, the ortholog of human *MYCN*, is expressed ubiquitously at high levels in the CNS before 24 hpf, but becomes restricted thereafter [55]. This could explain why proliferative effects of overexpressed Alk were higher at earlier embryonic stages.

### Alk is required for differentiation and survival in the CNS but not proliferation

We show that an *alk* knock-down does not affect cell proliferation in the hindbrain at 24 hpf. Interestingly, decreased cell proliferation was reported in *in vitro* cultured neuroblastoma cell lines where *ALK* has been depleted by siRNA [8], [11]. Also, a recent report using cultured chicken sympathetic neurons derived from PNS sympathetic ganglia showed that the knock-down of endogenous chicken *Alk* results in reduced proliferation [15]. This suggests possible differences of Alk function in different tissues and cell types. Alternatively, it could also reflect differences between *in vivo* and *in vitro* cell culture conditions suggesting more complicated scenarios that underlay Alk mediated cell cycle control, such as impact of the microenvironment or functional redundancy with other kinases that cannot be fully mimicked in cell culture settings.

In *alk* deficient embryos, generated by MO-mediated ALK depletion or pharmacological inhibition of its enzymatic activity, a universal increase in neural cell apoptosis and reduction in expression of selected proneural genes was observed. Since progenitor marker expression was unchanged and cell proliferation was not affected in hindbrain, this indicates that progenitor cells are not impaired by *alk* deficiency and that the observed apoptosis does not originate from neural progenitors. On the other hand, reduced proneural gene expression in differentiating neuronal precursors suggests defects in differentiation eventually leading to cell death. There are several possibilities how *alk* deficiency could affect cell survival. First, differentiating neural cells could undergo apoptosis because Alk is a survival factor and functions as a repressor of cell death. Thus, reduced proneural gene expression could simply reflect a reduction in the numbers of differentiating cells. Alternatively, Alk could be a specification factor, which is required to promote proneural gene expression. In morphants, cells that have exited the progenitor state would therefore fail to differentiate and consequently commence apoptosis. It remains unclear at present whether Alk induced apoptosis is the cause or consequence of impaired neurogenesis. However, our study showed that the extent of apoptosis in embryos deficient for Alk is relatively mild compared to the extensive changes in neurogenic gene expression. This observation favours a scenario where Alk deficiency leads to impaired neurogenesis, which subsequently results in apoptosis.

Unfortunately, Alk knock-out animals other than the embryos presented here are lacking in order to address which of the scenarios explains increased apoptosis after *alk* knock-down. Interestingly and in agreement with a role as specification factor, the Alk co-ortholog Ltk is involved in cell fate specification of multipotent precursors in the iridophore lineage (Lopes et al., 2008). On the other hand, siRNA-based approaches recently reported increased apoptosis upon Alk knock-down in neuroblastoma cell lines [56] supporting the idea of Alk as a survival factor. It is well established that proneural genes are required for cell survival during neuronal differentiation [57], [58], [59]. Based on this and our own observations, a combination of both scenarios therefore seems likely. We propose that Alk is required for the survival of neural precursors that have exited the progenitor state and are about to enter different neural differentiation fates.

## Supporting Information

Figure S1 **Sense controls and expression of zebrafish** ***alk*** **at 72 hpf.** **(A–D)** Negative controls at 24 hpf using a sense probe, corresponding to Fig. 1D–G, respectively. **(E–G)** Negative controls at 48 hpf using a sense probe, corresponding to Fig. 1I–K, respectively. **(H)** Lateral view of embryo at 72 hpf with dorsal up and anterior to the left. **(I)** Dorsal high magnification views of head region at 72 hpf. **(J)** Negative sense probe control of (I). Scale bars: A–G, 100 µm; H–J, 200 µm.
(JPG)

Figure S2 **Heat-shock inducible transgenic zebrafish lines.** **(A,B)** Diagrams illustrating transgenic cassettes for control line (A) and *alk* overexpression lines (B). The construct reported by Bajoghli et al. contains eight heat shock element repeats (HSE) with bidirectional promoter activity. This construct was engineered in such a way that *alk* and *cfp* were put on each side (B), or just *cfp* in the control line (A). **(C–E)** 24hpf embryos from the control line (HSE:*cfp*, C), *alk* overexpression line 1 (*alk*:HSE:*cfp* ^1^, D), and independent *alk* overexpression line 2 (*alk*:HSE:*cfp* ^2^, E). All embryos received a 1.5 hour long heat shock at 39.5°C and at 10.5 hpf. Bright field images on top and images of the same embryos in CFP channel below. Inserts show representative transgene positive embryos in each line. Arrows point to transgenic negative siblings in each line. They are morphologically normal and not visible in the CFP channel.
(JPG)

Figure S3 ***In situ*** **hybridization to detect** ***alk*** **overexpression in** ***alk*** **:HSE:** ***cfp*** **transgenic embryos.** A heat shock was performed at 24 hpf. All *in situ* hybridizations were done using an *alk* antisense RNA probe, at 1 hour post heat shock (hph) **(A)**, 6 hph **(B)**, 22 hph **(C)**, and shortly after a second HS performed at 48 hpf **(D)**. Inserts show representative transgenic positive embryos. As soon as 1 hour after heat shock, high levels of exogenous *alk* RNA could be detected in the entire embryo. Reduced staining at 6 hours after HS indicated RNA degradation. Staining almost completely disappeared at 22 hours after HS. A second HS re-activated transcription of overexpressed *alk*. Arrows points to transgene negative siblings where no exogenous *alk* was detected. Endogenous *alk* expression was not detected because of short staining time.
(JPG)

Figure S4 **Proliferation and differentiation in the** ***alk*** **:HSE:** ***cfp*** **^2^ line.** **(A,B)** Confocal sections of 24hpf embryos of the *alk*:HSE:*cfp* ^2^ line. Sib (A) and Tg+ (B) had different neural tube shapes. Dividing cells (pH 3, green) and neurons (HuC/D, red) in Tg+ embryos (B) were both mispositioned (arrowhead), identical to defects observed in the *alk*:HSE:*cfp* ^1^ line (Fig. 2). Asterisk labels 4^th^ ventricle. **(C,C′,D,D′)** Ectopic pERK (arrows) in Tg+ (D,D′) of the *alk*:HSE:*cfp* ^2^ line compared to Sib (C,C′), identical to defects observed in *alk*:HSE:*cfp* ^1^ (Fig. 3). pERK, phosphorylated-ERK1/2. Sib, transgene negative siblings. Tg+, transgenic embryos. Scale bars: 50 µm.
(JPG)

Figure S5 ***alk*** **MO efficiency test experiments.** **(A)** A schematic diagram showing arrangement of the first four *alk* exon-intron boundaries and MO binding sites. Numbers show exon/intron sizes in basepairs. **(B)** A construct containing *egfp* inserted in frame with the *alk* ATG site was generated to test the binding efficiency of ATG-MO. **(C–F)** Injection of this mRNA in combination with MOs resulted in different EGFP translation levels. Embryos at 8 hpf, with bright field images on top and fluorescent images below. Injection of the mRNA resulted in EGFP signal (D). Co-injection with *alk* ATG-MO blocked its translation (E). *alk* ATGmismatch-MO did not block its translation (F). Embryos labeled with asterisks in (C) and (E) were taken from the group shown in (D), indicating sufficient fluorescent excitation. **(G)** A diagram showing aberrant splice products with retention of intron 3, when *alk* pre-mRNA splicing is blocked by splicing MOs. **(H)** RT-PCRs were used to test splicing MO efficiency by primers indicated in (A). In Spl-MO injected 24 hpf embryos, the PCR product size was increased by approximately 300 bp (2^nd^ lane) when compared to uninjected wild-type (1^st^ lane), indicating retention of intron 3. -RT controls (3^rd^ and 4^th^ lanes, without reverse transcriptase in cDNA synthesis) excluded contamination by genomic DNA. *gapdh* was used as loading control.
(JPG)

Figure S6 **Increased apoptosis in** ***alk*** **morphants.** TUNEL staining of embryos at 22 hpf. Images show lateral views on top with anterior to the left, and dorsal views of head region below with anterior to the top. WT **(A)** or Std-MO injected **(B)** embryos had only few scattered TUNEL positive cells. In *alk* ATG-MO **(C)** or Spl-MO injected embryos **(F)**, TUNEL positive cells increased in number, while ATGmismatch-MO injected **(E)** looked normal. Co-injections with p53-MO **(D,G)** failed to attenuate the apoptosis defect indicating specificity of the effect. Scale bars: 100 µm.
(JPG)

Figure S7 **An** ***alk*** **ATG-MO leads to reduced proneural gene expression.** **(A,C,E,G,I,K)** Embryos injected with *alk* ATGmismatch-MO showed no difference when compared to WT in Fig. 5. **(B,D,F,H,J,L)** *alk* ATG-MO injected embryos show the same phenotype as *alk* Spl-MO injected embryos (Fig. 5). Embryos showed no change in *her6*, *neurog1* and *gfap* expression, but reduced expression of *neurod4, ascl1b* and *dla* identical to *alk* Spl-MO injected embryos shownin Fig. 5. Images show dorsal views of head regions with anterior to the left. Scale bars: 50 µm.
(JPG)
