# Supplementary figures and images for: Anaplastic Lymphoma Kinase Is Required for Neurogenesis in the Developing Central Nervous System of Zebrafish
Source: PLoS One. 2013 May 8;8(5):e63757. doi: 10.1371/journal.pone.0063757 (PMC3648509; doi:10.1371/journal.pone.0063757)

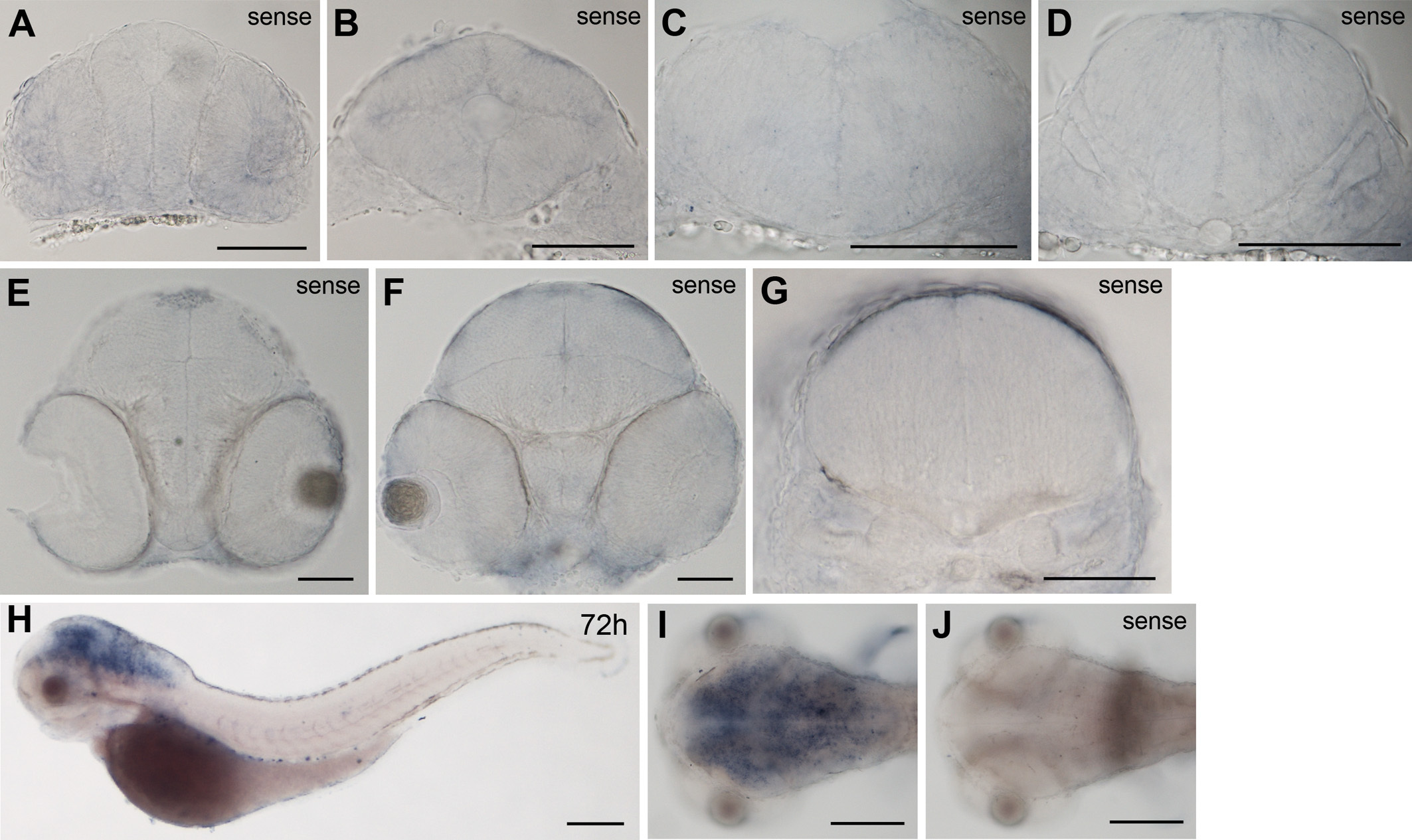

Supplement: Figure S1 — Sense controls and expression of zebrafish alk at 72 hpf. (A–D) Negative controls at 24 hpf using a sense probe, corresponding to Fig. 1D–G, respectively. (E–G) Negative controls at 48 hpf using a sense probe, corresponding to Fig. 1I–K, respectively. (H) Lateral view of embryo at 72 hpf with dorsal up and anterior to the left. (I) Dorsal high magnification views of head region at 72 hpf. (J) Negative sense probe control of (I). Scale bars: A–G, 100 µm; H–J, 200 µm. (JPG) [file pone.0063757.s001.jpg]

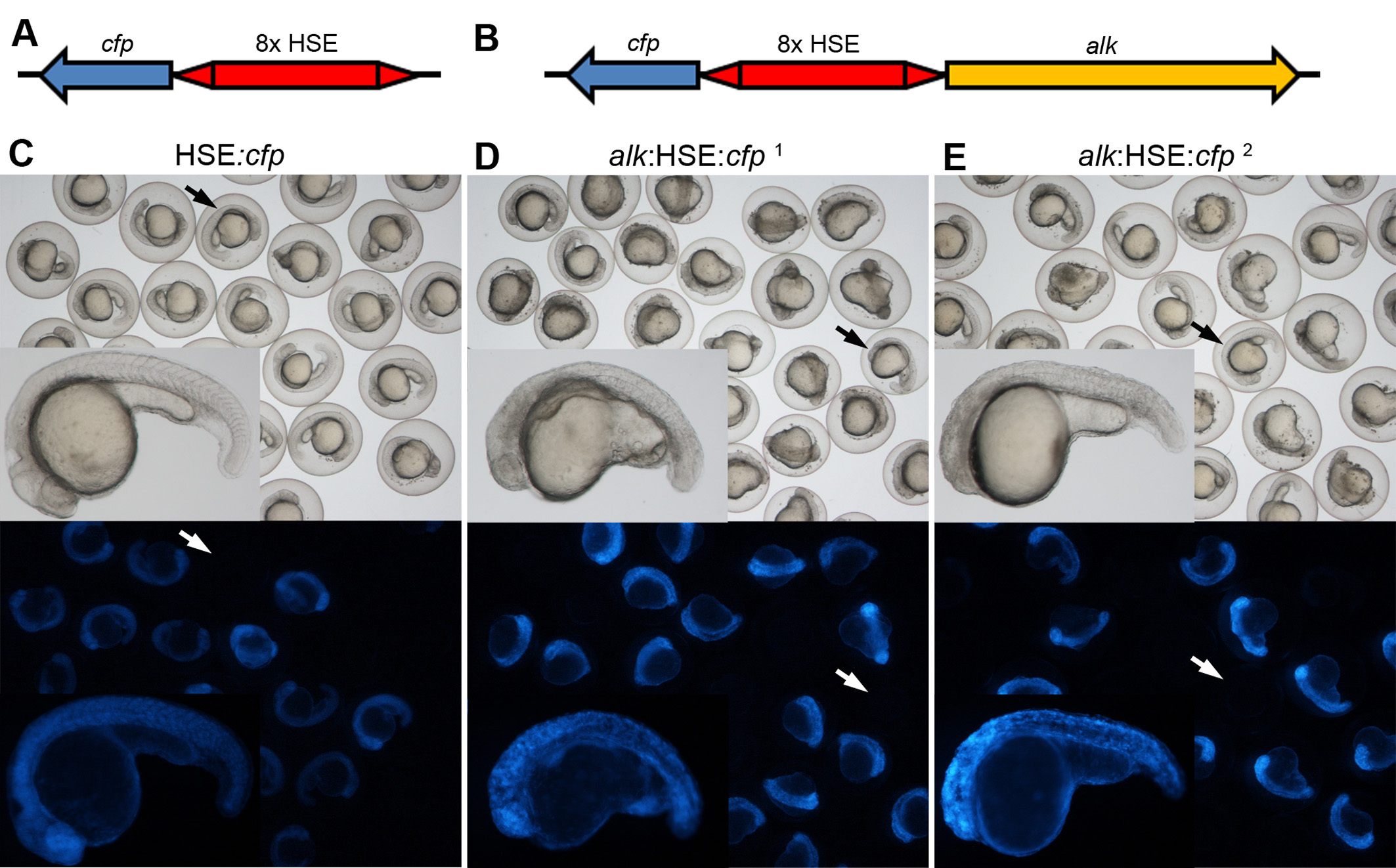

Supplement: Figure S2 — Heat-shock inducible transgenic zebrafish lines. (A,B) Diagrams illustrating transgenic cassettes for control line (A) and alk overexpression lines (B). The construct reported by Bajoghli et al. contains eight heat shock element repeats (HSE) with bidirectional promoter activity. This construct was engineered in such a way that alk and cfp were put on each side (B), or just cfp in the control line (A). (C–E) 24hpf embryos from the control line (HSE:cfp, C), alk overexpression line 1 (alk:HSE:cfp 1, D), and independent alk overexpression line 2 (alk:HSE:cfp 2, E). All embryos received a 1.5 hour long heat shock at 39.5°C and at 10.5 hpf. Bright field images on top and images of the same embryos in CFP channel below. Inserts show representative transgene positive embryos in each line. Arrows point to transgenic negative siblings in each line. They are morphologically normal and not visible in the CFP channel. (JPG) [file pone.0063757.s002.jpg]

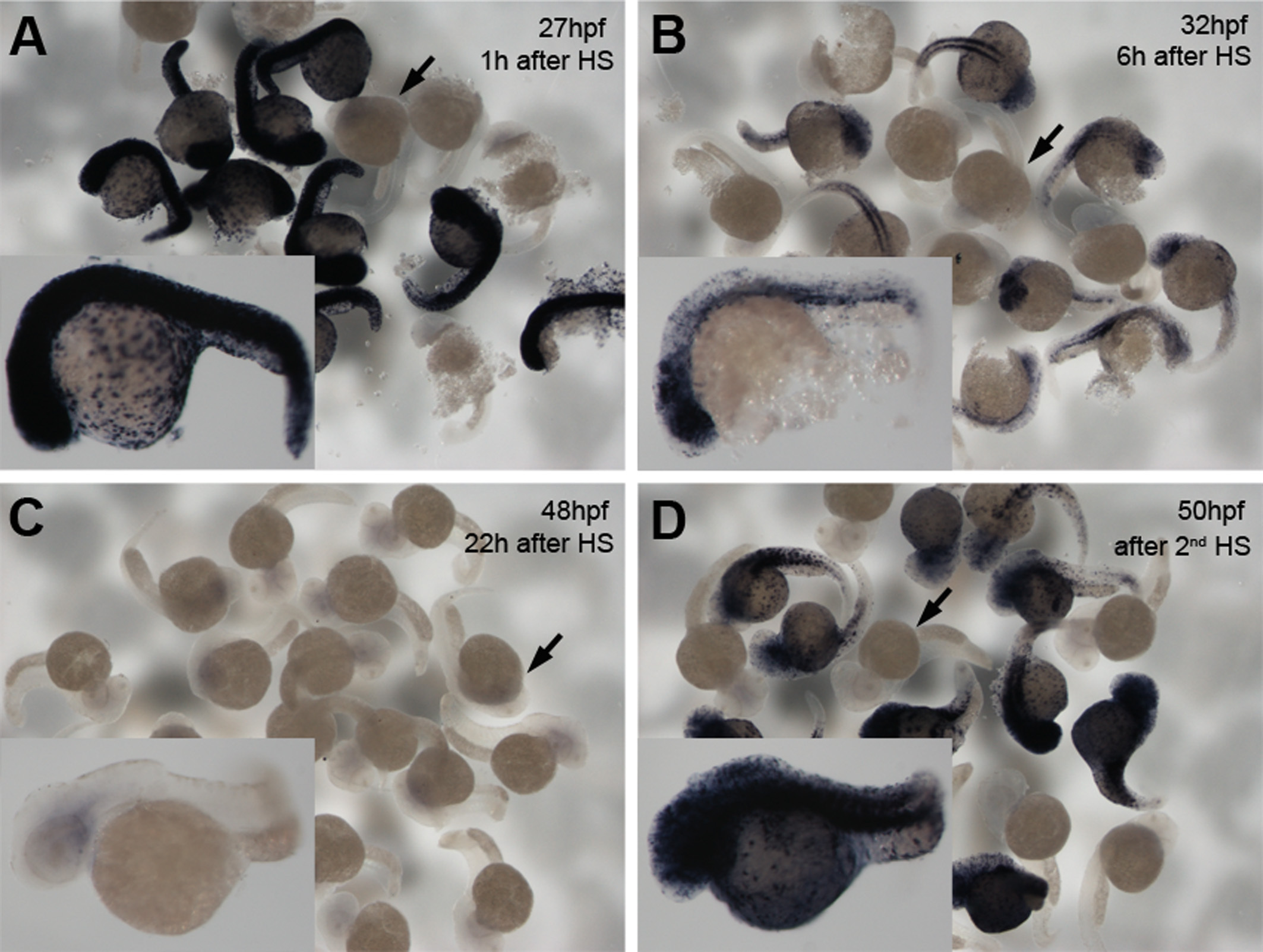

Supplement: Figure S3 — In situ hybridization to detect alk overexpression in alk :HSE: cfp transgenic embryos. A heat shock was performed at 24 hpf. All in situ hybridizations were done using an alk antisense RNA probe, at 1 hour post heat shock (hph) (A), 6 hph (B), 22 hph (C), and shortly after a second HS performed at 48 hpf (D). Inserts show representative transgenic positive embryos. As soon as 1 hour after heat shock, high levels of exogenous alk RNA could be detected in the entire embryo. Reduced staining at 6 hours after HS indicated RNA degradation. Staining almost completely disappeared at 22 hours after HS. A second HS re-activated transcription of overexpressed alk. Arrows points to transgene negative siblings where no exogenous alk was detected. Endogenous alk expression was not detected because of short staining time. (JPG) [file pone.0063757.s003.jpg]

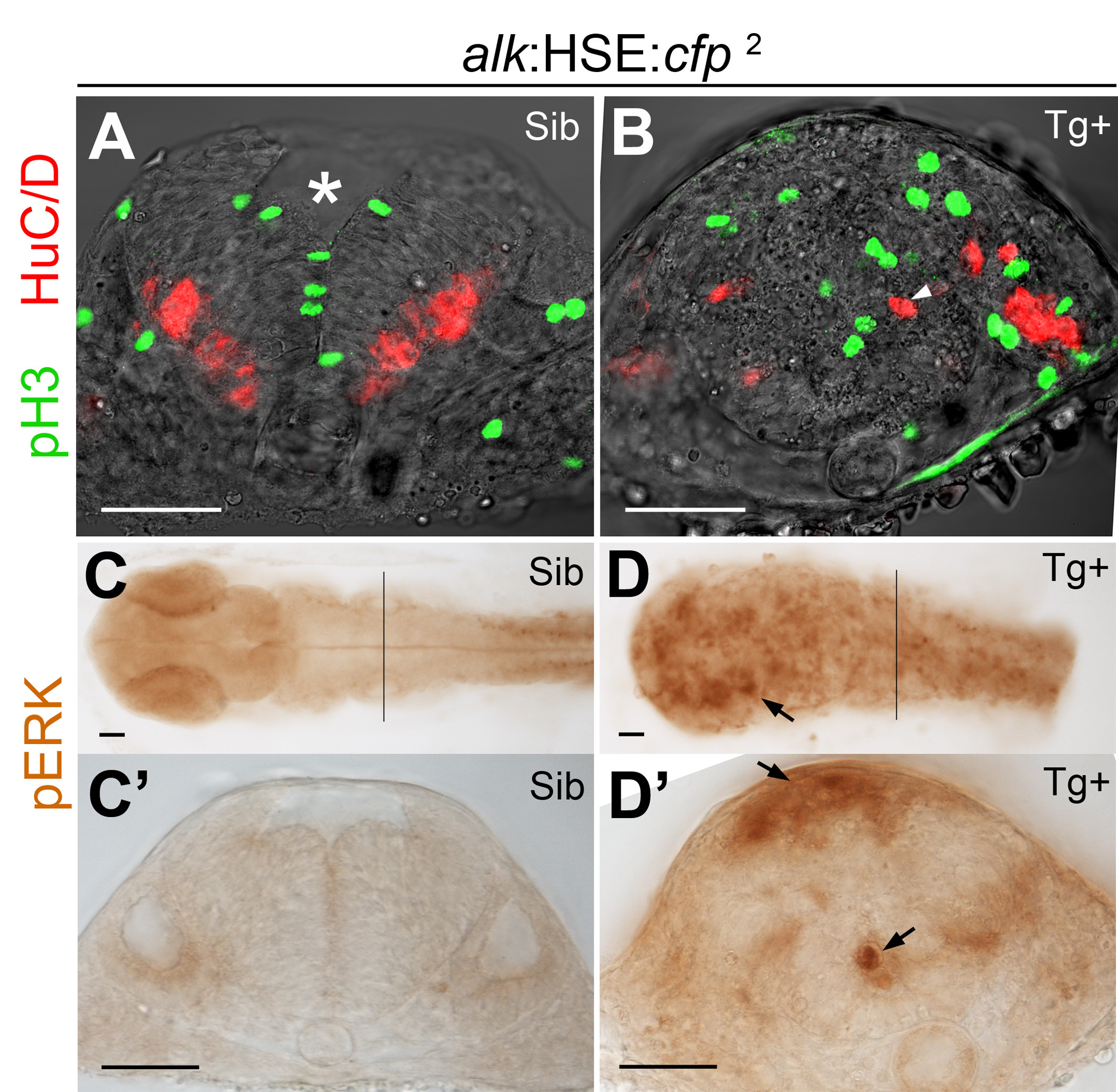

Supplement: Figure S4 — Proliferation and differentiation in the alk :HSE: cfp 2 line. (A,B) Confocal sections of 24hpf embryos of the alk:HSE:cfp 2 line. Sib (A) and Tg+ (B) had different neural tube shapes. Dividing cells (pH 3, green) and neurons (HuC/D, red) in Tg+ embryos (B) were both mispositioned (arrowhead), identical to defects observed in the alk:HSE:cfp 1 line (Fig. 2). Asterisk labels 4th ventricle. (C,C′,D,D′) Ectopic pERK (arrows) in Tg+ (D,D′) of the alk:HSE:cfp 2 line compared to Sib (C,C′), identical to defects observed in alk:HSE:cfp 1 (Fig. 3). pERK, phosphorylated-ERK1/2. Sib, transgene negative siblings. Tg+, transgenic embryos. Scale bars: 50 µm. (JPG) [file pone.0063757.s004.jpg]

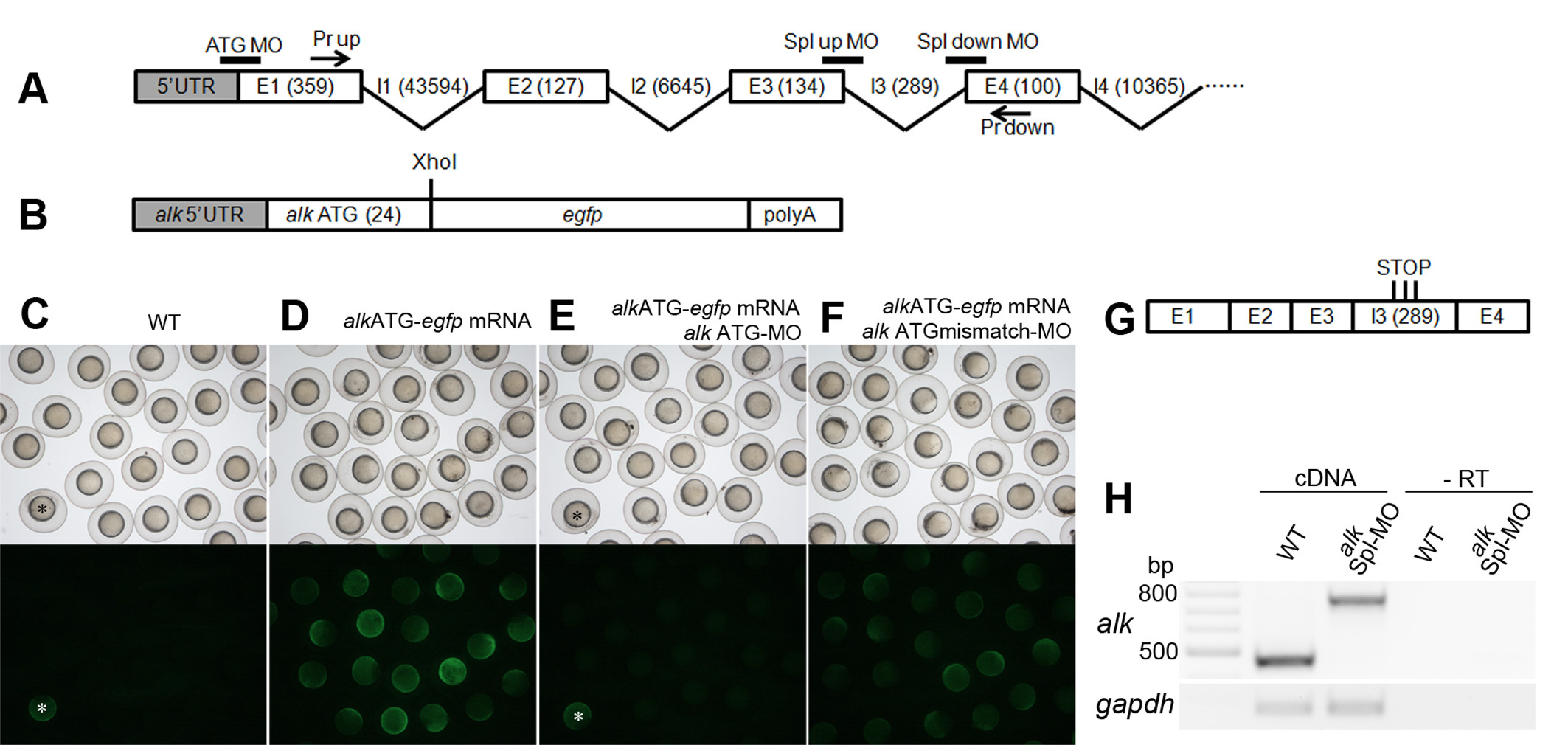

Supplement: Figure S5 — alk MO efficiency test experiments. (A) A schematic diagram showing arrangement of the first four alk exon-intron boundaries and MO binding sites. Numbers show exon/intron sizes in basepairs. (B) A construct containing egfp inserted in frame with the alk ATG site was generated to test the binding efficiency of ATG-MO. (C–F) Injection of this mRNA in combination with MOs resulted in different EGFP translation levels. Embryos at 8 hpf, with bright field images on top and fluorescent images below. Injection of the mRNA resulted in EGFP signal (D). Co-injection with alk ATG-MO blocked its translation (E). alk ATGmismatch-MO did not block its translation (F). Embryos labeled with asterisks in (C) and (E) were taken from the group shown in (D), indicating sufficient fluorescent excitation. (G) A diagram showing aberrant splice products with retention of intron 3, when alk pre-mRNA splicing is blocked by splicing MOs. (H) RT-PCRs were used to test splicing MO efficiency by primers indicated in (A). In Spl-MO injected 24 hpf embryos, the PCR product size was increased by approximately 300 bp (2nd lane) when compared to uninjected wild-type (1st lane), indicating retention of intron 3. -RT controls (3rd and 4th lanes, without reverse transcriptase in cDNA synthesis) excluded contamination by genomic DNA. gapdh was used as loading control. (JPG) [file pone.0063757.s005.jpg]

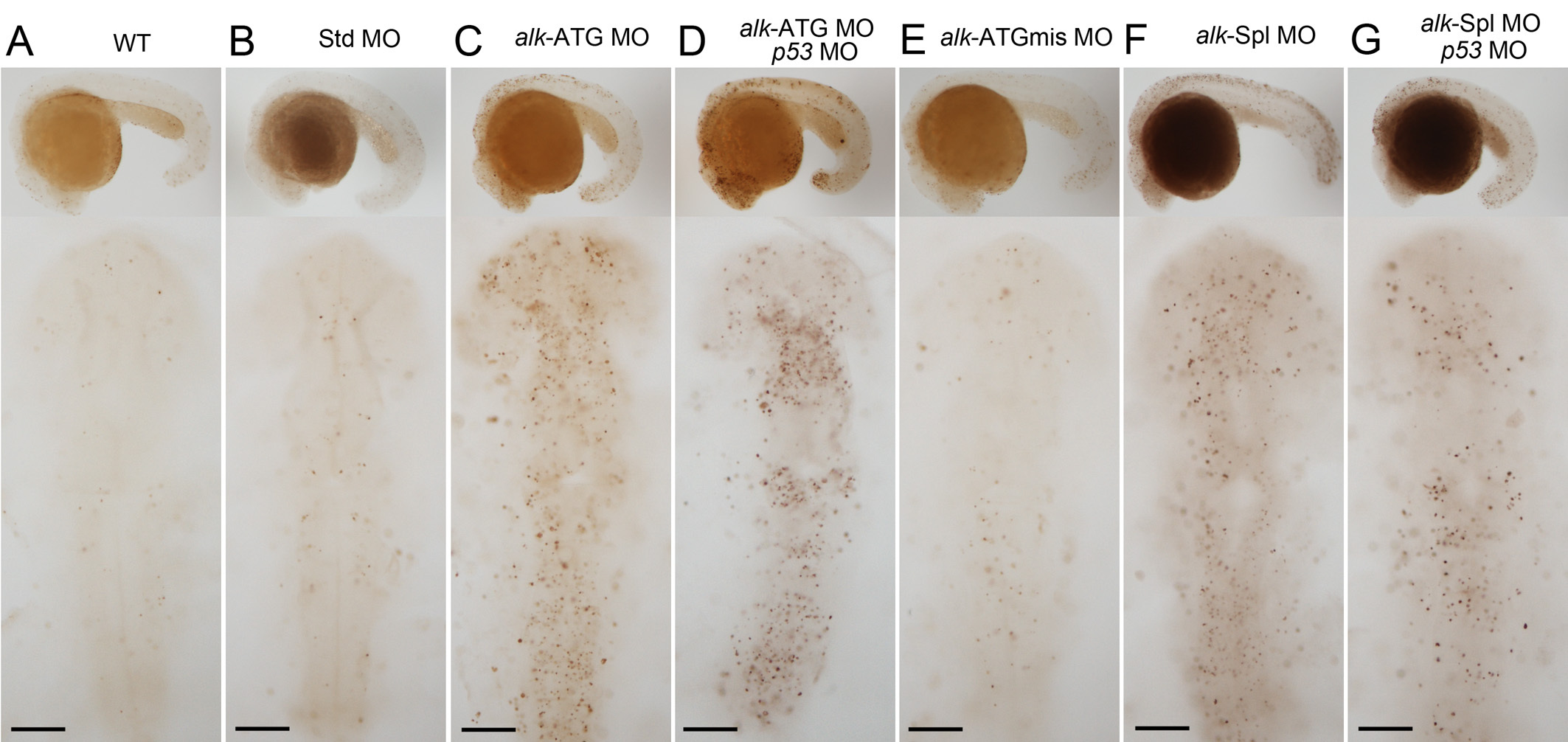

Supplement: Figure S6 — Increased apoptosis in alk morphants. TUNEL staining of embryos at 22 hpf. Images show lateral views on top with anterior to the left, and dorsal views of head region below with anterior to the top. WT (A) or Std-MO injected (B) embryos had only few scattered TUNEL positive cells. In alk ATG-MO (C) or Spl-MO injected embryos (F), TUNEL positive cells increased in number, while ATGmismatch-MO injected (E) looked normal. Co-injections with p53-MO (D,G) failed to attenuate the apoptosis defect indicating specificity of the effect. Scale bars: 100 µm. (JPG) [file pone.0063757.s006.jpg]

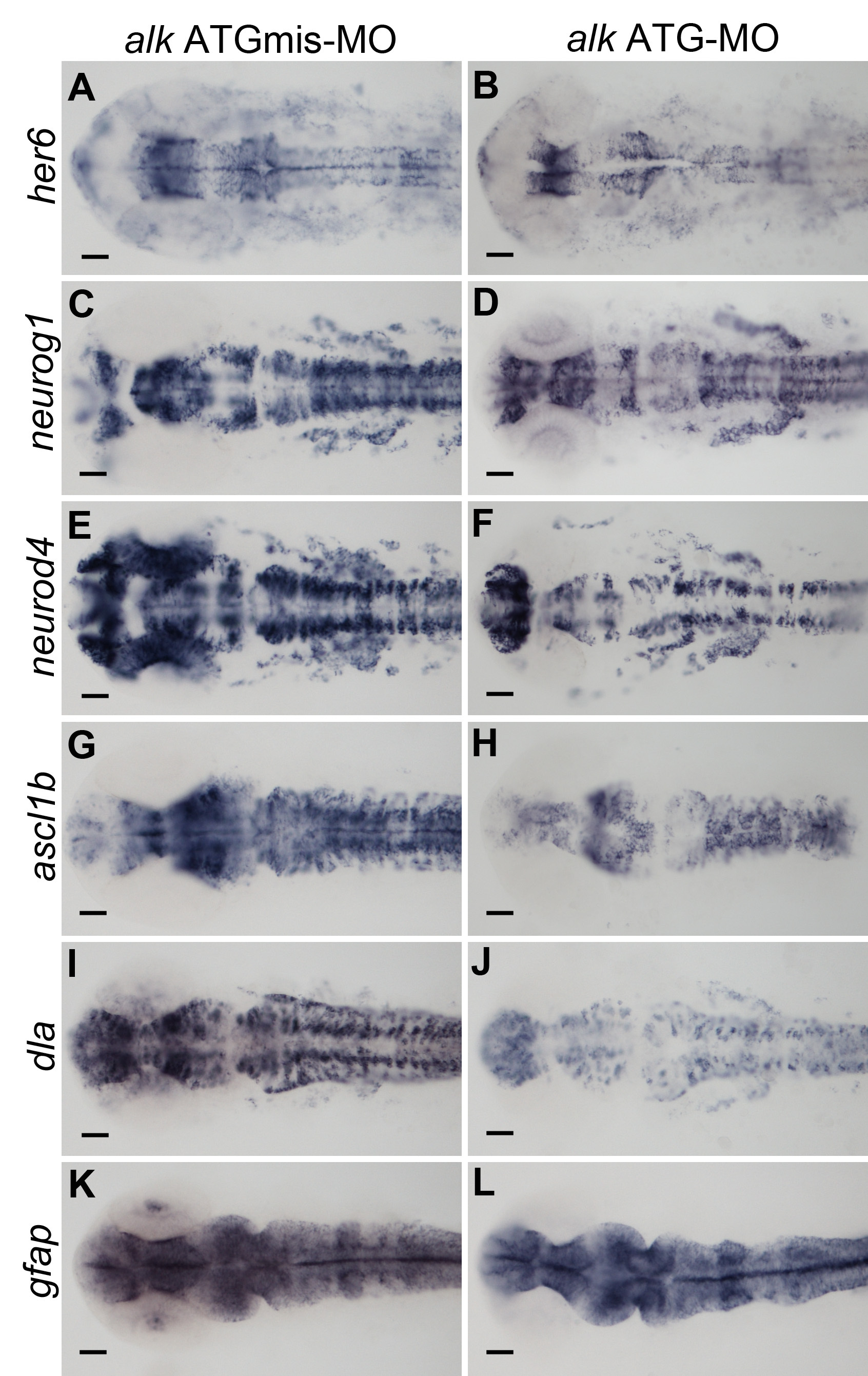

Supplement: Figure S7 — An alk ATG-MO leads to reduced proneural gene expression. (A,C,E,G,I,K) Embryos injected with alk ATGmismatch-MO showed no difference when compared to WT in Fig. 5. (B,D,F,H,J,L) alk ATG-MO injected embryos show the same phenotype as alk Spl-MO injected embryos (Fig. 5). Embryos showed no change in her6, neurog1 and gfap expression, but reduced expression of neurod4, ascl1b and dla identical to alk Spl-MO injected embryos shownin Fig. 5. Images show dorsal views of head regions with anterior to the left. Scale bars: 50 µm. (JPG) [file pone.0063757.s007.jpg]
